# Data on a thermostable enzymatic one-pot reaction for the production of a high-value compound from l-arabinose

**DOI:** 10.1016/j.dib.2018.05.140

**Published:** 2018-05-31

**Authors:** Maria Bawn, Fabiana Subrizi, Gary J. Lye, Tom D. Sheppard, Helen C. Hailes, John M. Ward

**Affiliations:** a The Advanced Centre for Biochemical Engineering, Department of Biochemical Engineering, University College London, Bernard Katz Building, London WC1E 6BT, UK; b Department of Chemistry, University College London, 20 Gordon Street, London WC1H 0AJ, UK

## Abstract

The dataset presented in this article is related to the research article entitled “One-pot, two-step transaminase and transketolase synthesis of l-*gluco*-heptulose from l-arabinose” (Bawn et al., 2018 in press) [1]. This article presents data on initial experiments that were carried out to investigate new thermostable transketolase (TK) activities with l-arabinose. Transaminase (TAm) sequences from an in-house library of thermophilic strains were analyzed to compare homologies to characterized TAms with desired activity. DNA and amino acid sequences are presented for all the enzymes investigated. Calibration curves for products of the TK and TAm reactions are also presented along with chromatographic analysis of the various one-pot reactions.

**Specifications Table**

**Table:** 

Subject area	*Biology*
More specific subject area	*Biocatalysis*
Type of data	*Tables, text file, figures*
How data was acquired	*Experiments/ in-vitro assays and high performance anion exchange chromatography with pulsed amperometric detection (HPAEC-PAD)*
Data format	*Analyzed and tabulated*
Experimental factors	*All enzymes and substrates were freshly prepared before use*
Experimental features	*Experiments were carried out in triplicate*
Data source location	*United Kingdom, London, University College London (UCL)*
Data accessibility	*The data are accessible only within this article*

**Value of the data**

• The data presented in this article gives new insight into the activities of thermostable enzymes not published before.
• The data represents a rationale behind why TKs and TAms were selected for the one-pot reaction.
• Product of one-pot reaction, l-*gluco*-heptulose, is a pharmaceutically-relevant compound.

## 1 Data

l-Arabinose is a major monosaccharide of sugar beet pulp (SBP), a by-product of sucrose extraction which is currently produced and sold as a low value animal feed [1]. The main focus of this work was to create a value-added product from the monosaccharides that make up SBP via enzymatic routes. Building on previous work [2], [3], this present study produces l-*gluco*-heptulose, a high value, pharmaceutically relevant compound from l-arabinose using a two-step thermostable enzyme cascade. A thermostable TK catalyzed the synthesis of l-*gluco*-heptulose from l-arabinose and β-hydroxypyruvate (HPA) in which the latter was produced *in situ* from l-serine and α-ketoglutaric acid using a thermostable TAm.

Table 1 identifies thermostable TKs utilized and whether they were active towards l-arabinose via the Seliwanoff assay [4]. Table 2 describes the TAms investigated and compares sequence homologies to TAms previously showing activities required for this reaction. Examples of HPAEC-PAD traces (Fig. 2, Fig. 3) demonstrate how the TK and TAm one-pot reactions were monitored for the presence of l-*gluco*-heptulose.

**Table 1:** TKs showing activity with l-arabinose via Seliwanoff assay.

**TK Strain**	**UniProtKB accession code**	**Plasmid name and abbreviated name**	**Active towards l-arabinose from Seliwanoff assay**
*Deinococcus geothermalis* DSM 11300	Q1IW07	pQR1758 (TK_Dgeo_)	✓
*Deinococcus radiodurans* DSM 20539	Q9RS71	pQR1759 (TK_Drad_)	✓
*Geobacillus stearothermophilus* DSM 22	KFL15812.1	pQR1743	✓
*Thermobifida fusca strain* YX	Q47ND4	pQR1744	✗
*Thermotoga maritima* DSM 3109	Q9X283	pQR1745	✗

**Table 2:** Sequence similarity values between new cloned TAms and DGEO_0713, SPAT and CV2025.

**TAm Strain**	**UniProtKB accession code**	**Plasmid name and abbreviated name**	**Homology to DGEO_0713**	**Homology to SPAT**	**Homology to CV2025**
			***Deinococcus geothermalis*****(%)**	***Sulfolobus solfataricus*****(%)**	***Chromobacterium violaceum*****(%)**
*Deinococcus radiodurans* DSM 20539	Q9RWP3	pQR1746	78	38	31
*Geobacillus stearothermophilus* DSM 22	Q59228	pQR1756 (TAm_Gste_)	25	38	40
*Thermobifida fusca* strain YX	Q47LH8	pQR1748	26	37	41
*Thermotoga maritima* DSM 3109	G4FE93	pQR1749	30	58	31
*Deinococcus geothermalis* DSM 11300	Q1IZC2	pQR1757 (TAm_Dgeo_)	29	29	44
*Xanthomonas campestris pv. Campestris* DSM 3586	Q8PDQ2	pQR1751	28	25	30
*Thermotoga maritima* DSM 3109	Q9X1C0	pQR1752	33	34	36
*Pectobacterium carotovorum subsp. Carotovorum* DSM 30168	A0A0B3YSH6	pQR1755	24	31	33

**Fig. 1:**
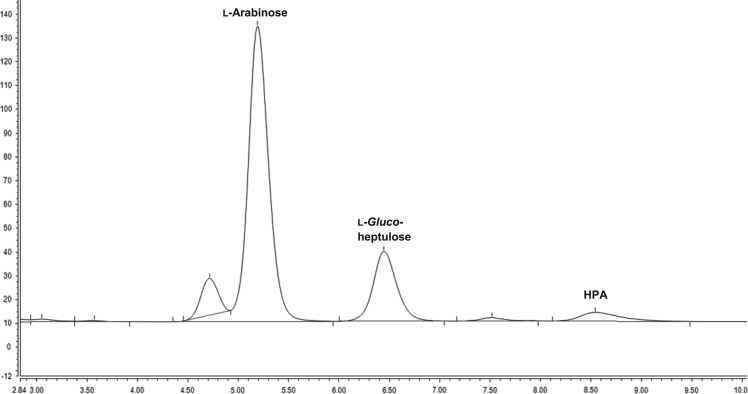
HPAEC-PAD trace showing the elution of l-arabinose, l-*gluco*-heptulose and HPA.

**Fig. 2:**
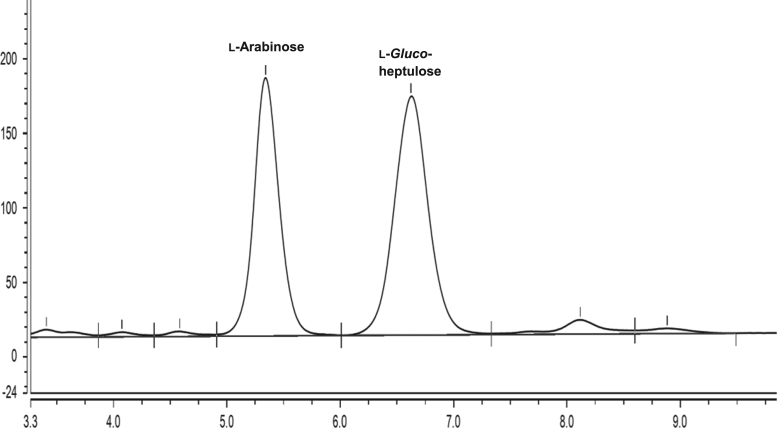
HPAEC-PAD trace showing l-*gluco*-heptulose production from one-pot reaction with TAm_Dgeo_ and TK_Dgeo_ after 24 h.

**Fig. 3:**
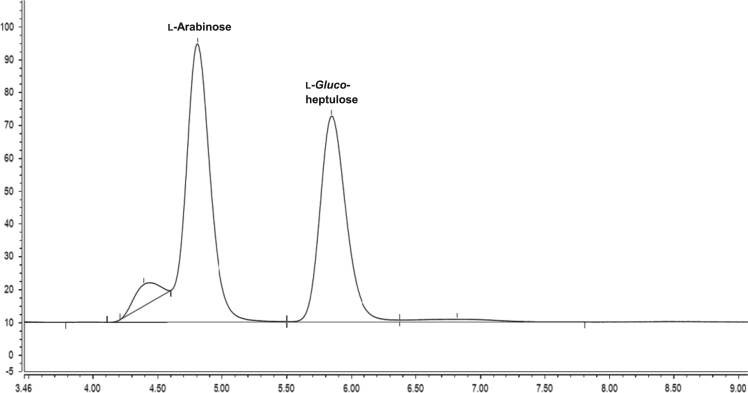
HPAEC-PAD trace showing l-*gluco*-heptulose production from one-pot reaction with TAm_Dgeo_ and TK_Drad_ after 24 h.

## 2 Experimental design, materials and methods

### 2.1 TK activity

Thermostable TKs were cloned and subsequently expressed in *E.coli* BL21 DE3. Cell lysates were used to determine activity towards l-arabinose using the colorimetric assay, Seliwanoff assay. The Seliwanoff assay distinguishes between ketoses and aldoses using 6 M HCl and resorcinol (Seliwanoff׳s reagent) [4]. After 24 h incubation of enzyme and l-arabinose, Seliwanoff reagent was added to the reaction and heated at 100 °C. Colour formation due to the presence of the ketose, l-*gluco*-heptulose, was observed within 15 min (Table 1).

### 2.2 TAm sequence analysis

TAm sequences were obtained from the NCBI database [5] and the UniProt Knowledgebase (UniProtKB) [6] followed by a sequence alignment using Clustal W [7].

### 2.3 Product analysis using HPAEC-PAD

**Fig. 4:**
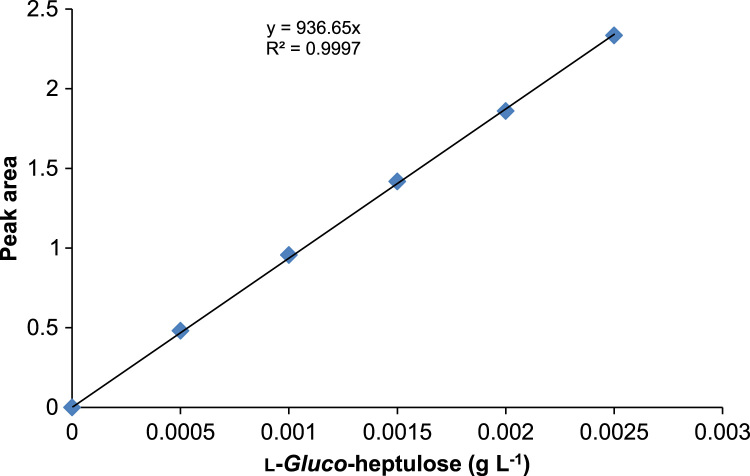
Calibration curve for determination of l-*gluco*-heptulose yield.

**Fig. 5:**
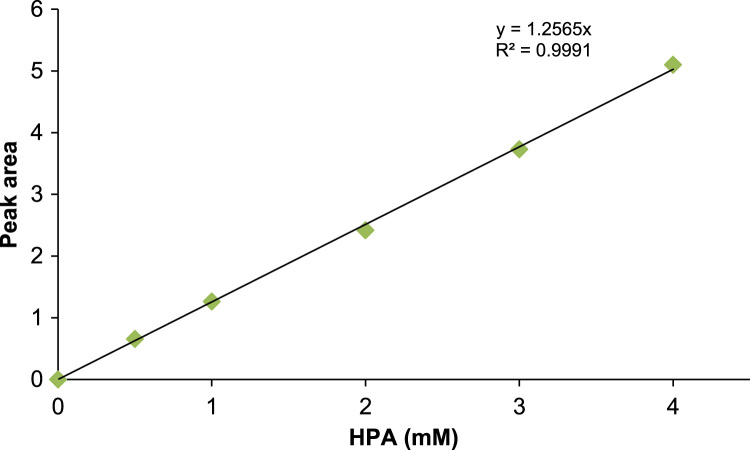
Calibration curve for determination of HPA yield.

Quantitative analysis of l-arabinose, l-*gluco*-heptulose and HPA was performed using HPAEC-PAD (ICS 5000+, Dionex) equipped with a Dionex Aminopac^TM^ PA1 anion exchange column 4×250 mm^2^ fitted with a Dionex Aminopac^TM^ PA1 guard column 4×50 mm^2^, an electrochemical detector system, and an eluent generator with a KOH 500 cartridge. The elution times of each compound can be observed in Fig. 1. Fig. 3, Fig. 4 are examples of a one-pot reaction analysis with various TKs and TAm_Dgeo_. Standard calibration curves of l-*gluco*-heptulose and HPA were used for quantification purposes (Fig. 4, Fig. 5).

### 2.4 TK DNA/ amino acid sequences

DNA sequences were retrieved from the NCBI database [5] and amino acid sequences were obtained through the UniProtKB [6].

**>TKDgeo**

ATGAGTCCCGAACAGCAGGCCGTGCGTCAGGATGTCGATCAGCTGAGCATCAACACCATCAGGACGCTTGCCATCGATGCGGTGCAGCGGGCCAACAGTGGCCACCCCGGCGCGCCGCTCGGCATGGCCCCGATGGGCTACGTGCTGTGGCAGCGCTTCCTGCGCCACAATCCGAAACATCCCGAGTGGCCGGGCCGCGACCGCTTCGTGCTGTCGGCAGGGCACGCCAGCATGCTGATCTACTCGCTGCTGCACCTCACCGGCTACGACCTGCCGCTGGAGGACATCAAGAACTTCCGCCAGTGGGGCAGCAAGACGCCTGGGCATCCCGAGTTCTTCCACACCCCAGGCCTAGACGCCACCACCGGCCCGCTCGGTCAGGGTGCGGCGATGACGGTGGGCATGGCGATGGCCGAAGCGCACCTCGCCGCACGCTACAACCGCCCCGGCTTCAAGGTCTTTGACAACTACACCTACGCGATCTTGGGGGACGGCGACCTGCAAGAAGGCGTCAACCACGAGGCCGCGTCGCTGGCAGGGCACCTCAAGCTGGGCAAGCTGATCTGGCTGCACGACGACAACCAGGTGCAGCTGGACACCGCCACGTTCAAGGCGGCCAACGAGGATACTGCGGAGCGTTACCGCGCCTACGGCTGGGAAGTTCTGCGTGTGCAAGACGGCAACAATCTCACGGAGATCGAGAACGCGATCCGCCAGGCACGGATGAACACCGAGCAGCCCACCCTGATCCAGGTTCGCACGGTGATCGGCTTCGGCAGTCCCCGTGCGGGCACCAGCAAGGCGCACGGCGAGCCGCTGGGCGAGGAAGGCGTGCAGGAGACCAAGGCGGCCCTGGGCTGGGACTACCCGCCCTTCACGGTGCCCGACGAGGTCAAGGCGCATATGGACGCGACTGAGCGTGGCGCGGAGTGGGAGGCCGACTGGAACGCGCTGATGGAGCGCTACCGTGCCGAGTACCCCGATCTCGCGGCGGAGGTTGACGCGCTGCTGGCGCGCGAACTGCCCGCCAATCTCGCCGAAGTGCTCCCCTCCTACGAAGTGGGCAGCAAGGCCATCGCCACCCGCAACGCGAGCGGTGAAGTCATCAATGCGCTGGCGCAGGTGGTGCCGGGCCTGATGGGGGGCAGTGCGGATCTCTCCGGCAGCACCAAAACCACCATCAAGGACGGCGGCGAGTTTCTGCCAGGAAACTACGGGGGCCGCAACGTCTACTTTGGCGTCCGCGAGTTTGGGATGGCCGCAGCGGGCAATGGCCTTTCGCTCTACGGAGGTGTTCGGCCCCTGGTGGGGACCTTCCTGGTGTTTGCGGACTACCTCAAGCCCGCCTTCCGCCTCTCCGCCCTTCAGTTCCAGCCGGTTACCTATGTCCTGACCCATGACTCCATTGGCCTGGGCGAAGACGGCCCAACCCACCAGCCTATTGAGCAGCTCGCCATGCTGCGCGCCGTGCCGGGTGCCCACGTGATTCGCCCCGCCGACGCCAACGAGACGGCGGCGGCCTGGCAGATGGCGCTGGAGTACGACAAGGGACCAACCGCTCTGGCCCTCTCCCGCCAGGATCTCCCAGTGCTGCCCCGCAACCACGCGGGCGTGAAGAAGGGCGCCTACGTGGTTCGCGACGCCGAAGGGGGGCCGGCACAGATCATCTTGATCGCCACCGGCTCGGAGGTGAGCCTGGCGCTGGATGCTGCCCAAGCGCTGGCGGAGGAAGGCATCCAGGCTCGGGTCGTCTCAATGCCCTGCATGGAAGTCTTCCGCCAGCAGGACGCCAGTTATCGGGACAGCGTGCTCACCCCCGGCGTGAAACGCGTGGCCATCGAGGCTGCCAGCCCGCTCCCCTGGTATGAGTGGGTGGGCTTTGACGGCGCGGTGATCGGAATGACCACCTTTGGCGCCTCGGCCCCAGCCAAAGTCCTCTTTGAGAAATTCGGCTTCAACGTGCCGAACGTCGTGCAGGTCGTCAAGGGCGTTTTGCAGAGGTGA

**>TKDgeo**

MSPEQQAVRQDVDQLSINTIRTLAIDAVQRANSGHPGAPLGMAPMGYVLWQRFLRHNPKHPEWPGRDRFVLSAGHASMLIYSLLHLTGYDLPLEDIKNFRQWGSKTPGHPEFFHTPGLDATTGPLGQGAAMTVGMAMAEAHLAARYNRPGFKVFDNYTYAILGDGDLQEGVNHEAASLAGHLKLGKLIWLHDDNQVQLDTATFKAANEDTAERYRAYGWEVLRVQDGNNLTEIENAIRQARMNTEQPTLIQVRTVIGFGSPRAGTSKAHGEPLGEEGVQETKAALGWDYPPFTVPDEVKAHMDATERGAEWEADWNALMERYRAEYPDLAAEVDALLARELPANLAEVLPSYEVGSKAIATRNASGEVINALAQVVPGLMGGSADLSGSTKTTIKDGGEFLPGNYGGRNVYFGVREFGMAAAGNGLSLYGGVRPLVGTFLVFADYLKPAFRLSALQFQPVTYVLTHDSIGLGEDGPTHQPIEQLAMLRAVPGAHVIRPADANETAAAWQMALEYDKGPTALALSRQDLPVLPRNHAGVKKGAYVVRDAEGGPAQIILIATGSEVSLALDAAQALAEEGIQARVVSMPCMEVFRQQDASYRDSVLTPGVKRVAIEAASPLPWYEWVGFDGAVIGMTTFGASAPAKVLFEKFGFNVPNVVQVVKGVLQR

**>TKDrad**

ATGACAGACCAGAGCGTTTCCCAAAACGTGGCGCGGCTGAGTGTGAACACCATTCGCACGCTCGCCATTGACGCGGTGCAGGCCGCCAACTCGGGCCACCCCGGTGCGCCGCTGGGCATGGCCCCGATGGGCTACGTGCTGTGGCACAAGTTCCTGCGCCACAACCCCGCGCACCCTGAGTGGCCGGGCCGCGACCGCTTCGTGCTGTCGGCGGGGCACGCCTCCATGCTGATCTACAGCCTGCTGCACCTGACCGGCTACCAGGAAATGACGCTCGACGACCTGCGCCACTTCCGGCAGTGGGGCTACCACACCCCCGGCCACCCCGAGTTTTTCCACACCAAGGGTCTGGACGCGACCACCGGCCCGCTTGGGCAGGGCGCGGCGATGACGGTGGGCATGGCGATGGCCGAAGCACACCTCGCCGCCCGCTACAACCGCGAAGGCTTTCCGATTTTCGACAACCGCACCTACGCCATCATGGGCGACGGCGATCTGCAAGAAGGCATCAACCACGAAGCCGCCGCGCTCGCCGGGCACCTGAAACTCGGCAAGCTGATCTGGCTGCACGACGACAACCACATCCAGCTCGACACGCCCACGAACAAGGCCGAGTCCGAGGACACCGCCGCCCGCTTCCGCGCCTATGGCTGGAACGTGCTGAAGGTGGAAGACGGCGACAATCTGGACGAAATTGAAAAGGCGATTGCCGAGGCCCGCAGCCAAAGCGAGCGGCCCACGCTGATTCAGGTGCGCACCATCATCGGCTTCGGCAGCCCGCGCGCCGGCACGAGCAAGGCGCACGGCGAGCCGCTCGGCGAAGAGGGCGTGGCCGAGACGAAGAAAGCGTTGGGCTGGGAGTACCCCGCTTTTACCGTGCCCGACGAAGTGGCTGCGCACATGAACGCTCGCGCTAAGGGTGCTCAACTCGAAGCCGACTGGGAAAAACTGATGGCCGACTACCGCACCGCGCACCCCGACCTCGGCAAGGAAGTGGACGCGCTGCTCGCCCGTGAACTGCCCGCCAACCTCGCCGACCTGCTGCCCAAGTACGAAGTCGGCGGCAAGGCGGCGGCCACCCGCAACGCGAGCGGCGAAGTCATCAACGCGCTGGCGAAGGTGCTTCCCGGTTTGATGGGCGGCAGCGCGGACCTCTCGGGCTCGACCAAGACCACCATCAAGGACGGCGGCGAGATGGAAGCGGGCACCATGGGCGGGCGCAACGTGCTGTTCGGCGTGCGCGAGTTCGGCATGAGCGCCGCGGGCAATGGCCTGAGCCTCTACGGCGGCCTGCACCCGATGGTAGGCACCTTCCTGGTATTCGCCGACTACCTCAAGCCGGCTTTCCGCCTCTCGGCGCTGCAAATGCAGCCGGTGACTTACGTGCTGACCCACGACTCCATCGGTCTGGGCGAAGACGGGCCGACCCACCAGCCGGTGGACCAGCTCGCCATGCTGCGAGCGGTGCCGGGCGCCCACGTCATTCGCCCCGCCGACGCCAACGAAACCGCCGCCGCGTGGCTGATGGCGCTGGAATACGACAAGGGCCCCACCGCGCTCGCCCTCTCGCGCCAGGATCTGCCGATTCTGCCCGCCAACATCGAAGGCGTGAAGAAGGGCGCGTATGTCCTCCGAGATGTGGACGGTGCCGATGGTCAGGGGGCTCAAGTCATCCTGATC

GCCAGCGGCTCGGAAGTCGCCCTGGCCCTGAGCAGCGCCGAGCGGCTGGCCGAAGAGGGCGTGCAGGCCCGCGTGGTGTCCATGCCGTGCATGGAGGTCTTTCGCCAGCAGGAGCAGAGCTACCGCGACAGCGTGCTGACCCCCGGCGTGAAGCGCGTCGCCATCGAGGCCGCCAGCCCGCAGCCCTGGTACGAGTGGACGCTCGGCGGCCCAGTCATCGGCATGACGACCTTCGGTGCGTCGGCCCCGGCCAAGGTGCTGTTTGAGAAGTTCGGCTTCAGCGTGGAAAACGTGGTGAAGGTGGTCCACTCCGTGCTGTAA

**>TKDrad**

MTDQSVSQNVARLSVNTIRTLAIDAVQAANSGHPGAPLGMAPMGYVLWHKFLRHNPAHPEWPGRDRFVLSAGHASMLIYSLLHLTGYQEMTLDDLRHFRQWGYHTPGHPEFFHTKGLDATTGPLGQGAAMTVGMAMAEAHLAARYNREGFPIFDNRTYAIMGDGDLQEGINHEAAALAGHLKLGKLIWLHDDNHIQLDTPTNKAESEDTAARFRAYGWNVLKVEDGDNLDEIEKAIAEARSQSERPTLIQVRTIIGFGSPRAGTSKAHGEPLGEEGVAETKKALGWEYPAFTVPDEVAAHMNARAKGAQLEADWEKLMADYRTAHPDLGKEVDALLARELPANLADLLPKYEVGGKAAATRNASGEVINALAKVLPGLMGGSADLSGSTKTTIKDGGEMEAGTMGGRNVLFGVREFGMSAAGNGLSLYGGLHPMVGTFLVFADYLKPAFRLSALQMQPVTYVLTHDSIGLGEDGPTHQPVDQLAMLRAVPGAHVIRPADANETAAAWLMALEYDKGPTALALSRQDLPILPANIEGVKKGAYVLRDVDGADGQGAQVILIASGSEVALALSSAERLAEEGVQARVVSMPCMEVFRQQEQSYRDSVLTPGVKRVAIEAASPQPWYEWTLGGPVIGMTTFGASAPAKVLFEKFGFSVENVVKVVHSVL

**>pQR1743**

ATGGCGCATTCGATCGAAGAATTAGCGATTACCACCATTCGAACGCTGTCGATTGACGCGATCGAAAAAGCGAAATCCGGGCACCCGGGCATGCCGATGGGCGCGGCCCCGATGGCGTATACGCTCTGGACGAAATTTATGAACCATAATCCAGCGAATCCCAACTGGTTTAACCGCGACCGGTTTGTTTTGTCCGCTGGGCACGGGTCGATGCTGCTTTACAGCCTGCTTCATCTAAGCGGCTACGATGTCACGATGGACGACTTGAAACAGTTCCGCCAATGGGGAAGCAAAACGCCGGGCCATCCGGAATACGGCCATACGCCAGGGGTGGAGGCAACGACCGGCCCGCTCGGCCAAGGGATTGCGATGGCGGTCGGCATGGCGATGGCGGAACGGCATTTGGCGGCTGCATACAATCGCGATGGATTTGACATTATCAACCACTACACGTATGCGATTTGCGGCGACGGCGATTTGATGGAAGGAGTGGCGAGCGAAGCGGCGTCACTCGCCGGCCACTTGAAGCTCGGCCGTCTGATCGTCCTGTATGACTCGAACGACATTTCGCTGGACGGCGAGCTCAACTTGTCGTTTTCGGAAAACGTCGCCCAACGTTTCCAAGCGTACGGCTGGCAATATTTGCGCGTTGAGGACGGCAACAATATTGAAGAAATCGCCAAAGCGCTCGAGGAGGCGCGGACGGACCTCAGCCGGCCGACGCTCATTGAAGTAAAAACGACGATTGGCTACGGCGCGCCAAATAAAGCGGGCACGTCCGGCGTCCACGGCGCTCCGCTCGGCGCCCAAGAGGCGAAGTTGACGAAAGAAGCGTACCGCTGGACGTTTTCCGAAGATTTCTACGTGCCGGATGAAGTGTACGCTCATTTCCGGGAAACGGTGCAAGAAGCCGGAGCGAGAAAAGAAGCGGAGTGGAATGAGCGCTTCGTTGCTTACGAGCGGGCGCATCCGGAATTGGCCGCCGAGCTGAAGCAGGCGATTGAAGGGAAGCTTCCGGATGGCTGGGAAACATCGCTGCCGGTGTATGAAGCGGGCAAAAGCTTGGCGACCCGCTCATCGTCCGGGGAAGTGATCAACGCCATCGCCAAAGCGGTGCCGCATTGTTTGGCGGTTCGGCGGACTTGGCAAGCTCGAATAAAACGCTTATCAAAGGCGGCGGCAACTTCTTGCCGGACAGCTACGAAGGGCGCAACATTTGGTTTGGCGTGCGCGAGTTTGCCATGGGCGCGGCGTTAAACGGCATGGCGCTTCACGGCGGGTTGAAAGTGTTCGGCGGCACGTTCTTCGTGTTCTCCGACTACTTGCGCCCGGCGATTCGGCTGGCGGCGCTCATGGGCTTGCCGGTGACGTACGTGCTGACGCACGACAGCATCGCCGTCGGGGAAGACGGCCCGACGCATGAGCCGGTCGAGCATCTCGCTTCACTTCGGGCGATGCCGAACTTGTCAGTCATCCGGCCGGCTGACGCAAACGAAACGGCGGCCGCCTGGCGGCTGGCGCTCGAGTCGACGAACAAGCCGACTGCGCTCGTCTTGACGCGTCAAGATGTGCCGACATTGCCGACAACCGCTCAGTTGGCGTATGAAGGCGTGAAAAAAGGCGCGTACGTCGTTTCACCGGCGAAAAACGGCGCTCCGGAGGCGCTGTTGTTGGCGACTGGCTCGGAAGTCGGTCTGGCCGTCAAAGCGCAAGAAGCGCTCGCCGCTGAGGGCATCCATGTCTCCGTCATCAGCATGCCATCGTGGGACCGCTTCGAAGCGCAGCCAAAATCGTACCGCGATGAAGTGCTGCCGCCGGCCGTGACGAAGCGGCTCGCCATTGAAATGGGCGCCTCGCTCGGTTGGGAGCGCTACGTCGGCGCCGAGGGCGACATTTTGGCCATCGACCGATTCGGTGCTTCCGCTCCGGGAGAGAAAATCATGGCCGAGTATGGCTTTACGGTTGACAACGTCGTCCGCCGCACAAAAGCGCTGCTCGGCAAGTAA

**> pQR1743**

MAHSIEELAITTIRTLSIDAIEKAKSGHPGMPMGAAPMAYTLWTKFMNHNPANPNWFNRDRFVLSAGHGSMLLYSLLHLSGYDVTMDDLKQFRQWGSKTPGHPEYGHTPGVEATTGPLGQGIAMAVGMAMAERHLAAAYNRDGFDIINHYTYAICGDGDLMEGVASEAASLAGHLKLGRLIVLYDSNDISLDGELNLSFSENVAQRFQAYGWQYLRVEDGNNIEEIAKALEEARTDLSRPTLIEVKTTIGYGAPNKAGTSGVHGAPLGAQEAKLTKEAYRWTFSEDFYVPDEVYAHFRETVQEAGARKEAEWNERFVAYERAHPELAAELKQAIEGKLPDGWETSLPVYEAGKSLATRSSSGEVINAIAKAVPQLFGGSADLASSNKTLIKGGGNFLPDSYEGRNIWFGVREFAMGAALNGMALHGGLKVFGGTFFVFSDYLRPAIRLAALMGLPVTYVLTHDSIAVGEDGPTHEPVEHLASLRAMPNLSVIRPADANETAAAWRLALESTNKPTALVLTRQDVPTLPTTAQLAYEGVKKGAYVVSPAKNGAPEALLLATGSEVGLAVKAQEALAAEGIHVSVISMPSWDRFEAQPKSYRDEVLPPAVTKRLAIEMGASLGWERYVGAEGDILAIDRFGASAPGEKIMAEYGFTVDNVVRRTKALLGK

**>pQR1744**

ATGAACACCGGCACCCCAAAGACCCTGGACTGGTCTGATCTCGATAGACGTACCGTAGACGTGGTTCGTGCCCTGGCGATGGACGCGGTCGAAGAAGCGGGATCCGGGCACCCTGGAACCGCGATGAGTCTGGCGCCTGTGGCCTACCTGCTCTTCCAGAAGGTGATGCGGCACGATCCGACAGATCCGAAGTGGATCGGCCGCGACCGCTTCGTCCTGTCCTGCGGGCACTCCAGCCTCACGCTCTACATCCAGCTCTACCTGGCTGGCTACGGGCTGAGCCTGAACGACATCAAGCGGCTGCGCCAGTGGGGCAGCCTCACCCCGGGCCACCCCGAATACGGGCACACCGCCGGGGTGGAAACCACCACCGGCCCCTTGGGGCAGGGCATCGGCAACGCGGTCGGCATGGCCATGGCCGCCCGCCGGGAGCGGGGCCTGTTCGACCCGGACACCCCGATCGGGGAAAGCCCGTTCGACCACTACATCTACGTCCTGTGCTCTGACGGCGACGTCCAGGAGGGCATCAGCCACGAAGTAAGTGCCCTCGCCGGCACGCAGAAGCTCGGCAACCTCATCGTCATCTGGGACGACAACCGCATCTCCATCGAAGACGACACCCAGATCGCATTCACCGAAGACGTCGTCGCCCGCTACGCCGCCTACGGCTGGCACGTCCAAGAGGTCGAGTGGGTCGGCGAGGACGGCTCCTACCACGAAGACGTGGCGGCGCTGTACGACGCGATCCGGGCCGCCCAGGCGGAGACGGAACGTCCCTCTTTCATCCGGCTGCGCACCATCATCGGCTGGCCGTCCCCGAACAAGCAGAACACGGGGGCGATCCACGGCGCCGCGCTGGGGGCTGAAGAGGTCGCCGCCACCAAGCGGGTGCTGGGCCTCAACCCTGAGGCGCAGTTCGACGTGCCCAACGAGCTGCTGGAGCACGCCCGGGGCGTGGTGGCGCGGGGCCGCGCCGCCCGCCAGGAATGGGAGGCCTTGTTCGCCAAGTGGCGGGCCAACGCGGGCGAGCGTGCCGAACTGTTCGACCGGCTGATGGCAGGCTCGCTCCCGGACGGTTGGGAGAAGGCGATCCCGACCTTCGAGCCCAGCGCTAAGGGCATGGCCACCCGGAAAGCGTCCGGTGAGGTGCTGAGCGCGATCGCCCCGGTGCTGCCGGAGCTGTGGGGCGGCTCGGCGGACTTGGCCGGATCCAACAACACCACGCCTAAGGGCGAGCCGTCGTTCATCCCCGAGGAGCGGTCCACGAAGGCGTTCTCCGGCCACCGCTACGGCCGGGTGCTGCACTTCGGGATCCGTGAACACGGCATGGGGGCGATCCTCAACGGGATCGCGCTGCACGGCCCCACCCGCCCCTACGGTGGCACCTTCCTCGTGTTCAGCGACTACATGCGGCCGTCGGTGCGGCTGGCTGCCCTGATGAAGCTGCCGGTCACGTACGTGTGGACCCACGACTCGATCGGTCTGGGCGAAGACGGACCCACCCACCAGCCGGTGGAGCACCTGTGGTCGCTGCGCGCCATCCCCGGCCTGGCGGTGGTGCGTCCCGCCGACGCCAACGAGACGGCAGTGGCCTGGCGCACCATCCTGGAACGCAATGACGGCCCGGTGGCGCTCGCGCTGACCCGGCAGTCGGTTCCGGTTCTGGACCGCTCCGAGCTCGCCTCTGCGGAGCTGGTCTCCCGCGGCGGGTACATCCTGGCCGAAGCCAGCAACGGCCGTCCGGAGGCGATCATCATCGCCACCGGAAGTGAGGTGCAGATCGCGTTGGAGGCGCGTTCCCGCCTGGAGGAGTCGGGTACTCCTACCCGTGTGGTGTCGATGCCGTGCCTGGAGTGGTTCAACGAGCAGGACGACGCCTACCGCCAGCAGGTGCTTCCACCGTCGGTCCGGGTCCGGGTCTCCGTGGAAGCCGGGGTCGCCTTGGGCTGGCGCGAGCTGGTGGGCGAGTATGGCGAGTCGGTGAGTCTGGAACACTTCGGCGCTTCGGCTCCGTACGCGACTCTCTACGAGCAGTTCGGGCTCACCGCCGACCGGGTAGTGGCAGCCGTACACTCCAGCGCTGCCAAGCTCGGCGGTGACCGTGGATCAACGACCGGCAACTGA

**>pQR1744**

MNTGTPKTLDWSDLDRRTVDVVRALAMDAVEEAGSGHPGTAMSLAPVAYLLFQKVMRHDPTDPKWIGRDRFVLSCGHSSLTLYIQLYLAGYGLSLNDIKRLRQWGSLTPGHPEYGHTAGVETTTGPLGQGIGNAVGMAMAARRERGLFDPDTPIGESPFDHYIYVLCSDGDVQEGISHEVSALAGTQKLGNLIVIWDDNRISIEDDTQIAFTEDVVARYAAYGWHVQEVEWVGEDGSYHEDVAALYDAIRAAQAETERPSFIRLRTIIGWPSPNKQNTGAIHGAALGAEEVAATKRVLGLNPEAQFDVPNELLEHARGVVARGRAARQEWEALFAKWRANAGERAELFDRLMAGSLPDGWEKAIPTFEPSAKGMATRKASGEVLSAIAPVLPELWGGSADLAGSNNTTPKGEPSFIPEERSTKAFSGHRYGRVLHFGIREHGMGAILNGIALHGPTRPYGGTFLVFSDYMRPSVRLAALMKLPVTYVWTHDSIGLGEDGPTHQPVEHLWSLRAIPGLAVVRPADANETAVAWRTILERNDGPVALALTRQSVPVLDRSELASAELVSRGGYILAEASNGRPEAIIIATGSEVQIALEARSRLEESGTPTRVVSMPCLEWFNEQDDAYRQQVLPPSVRVRVSVEAGVALGWRELVGEYGESVSLEHFGASAPYATLYEQFGLTADRVVAAVHSSAAKLGGDRGSTTGN

**>pQR1745**

ATGGAAAGGTTTCCCTATGAAAAACTTCCAGAAAGCGAACTCAAAGAGTTGAAAGAACTCGGAAGGCTCTGCCGTGGCGACATACTGAAAATGACCTACATAGCTAACTCAGGCCATCCTGGAGGATCCATGTCTTCGATCGATCTTTATCTTACCGTCTTCAAGTACGCAAAACTCAGACCCGTCGATGATCCTGCAAGAGACAGAATCGTGATCAGCCATGGACACACTTCTCCGGGTGTCTACGCAGCTATGGCTCGTTTGGGGTTTGTCGATCTCGATGAAGTCCTCGCAGGATTCAGACACCCCGCTTCCGTTTTTGAAGGACACGTGACCCGAGGTGTTGGGATCATCGACTGGACAACCGGAAACCTCGGTCAGGGTCTTTCAGCCGGACTCGGTTTTGCCCTCGCATCCAGGTTCACAGGAAAAGATTACCACGTCTTTGTTCTCATGAGTGACGCAGAACAGGCAAAAGGACAGGTGGCGGAGGCAAGAAGAGTGGCGAAAAAGTACGGTGTCACGAATCTCACAGTGATCATCGACTACAACGACGCCCAGATCAGTGGCCGTGCCAGAGACGTCATGCCCGTGAACATAAAGGAAAACTACTTAGCGGACGGCTGGAGGGTCATCGAGATCGATGGGCACGACTACGAACAGATCTATCTCGCACTGAAAGAAGCGGTAGAAGACGAAC

TGAATCCCGTTGCCATAATCGCCAAAACGGTCATGGGAAAAGGCGTATCTTTCATGGAAAACGAGGTGAAATACCACGGAAAGCCTTTGAACAGAGAAGAACTCGAAAAAGCCCTCGCGGAACTCGGAATTGAAAACGATGTTGATGTGTACATCGAAAAAAGAAAACAACTTCCAGTGGAAAAACACAAGAAAGTCTACAAAACTTACCCGATCAAGATCGACACGGGAGAGCCCATCACCTACACCTCACCCACTGACAACAGAAGCGCATTCGGAAAAGCTATTCTGGATCTGGTGAAGAAGAACGTAAACAATCCAGAAACCACACCCATCGTCGCTGTGGACTGCGACCTGAAGGGATCGGTCAAACTCGACCTGCTCGACAAAGAGTTCCCTGAGAGACTCCTGGAAGTGGGCGTTCAGGAACACAACGCTGCCGCTATGGCGGGGGCACTCTCCGCAGAGGGTGTGATCACGTTCTTCGCTGATTTTGGTGTTTTTGGAATTTCTGAAACCTACAACCAGCACAGGCTGAACGCCATCAATGGAACGAACCTCAAAGTCGTTGTCACACACTGCGGACTCAACGTGGGAGAGGACGGAAAAACTCATCACGGACTCGACTACGTTTCCGGGCCGATGAACTGGTACGGTTTCAAAGTGATCGTCCCTGGTGATCCCAACCAGACGGATAGAGTTGTCAGATACGCCGCGAAGGAATACGGGAACTTCGTAATCGCCATGGGAAGATCTAAGCTTCCCATCATCCTCGATGAAAACGGGAAACCTTTCTTCGGAGAGGGTTACACCTTCGAATATGGGAAGATCGATGTCGTTAGAAAAGGTGACGACGCGGTGATCATAACTTACGGTTCTACACTCTGTGAAGCCGTAAATGCCGCAGACGAACTCAAGAAAGAAGGAGTAAACGTAGCCGTTCTGAATGTCTCCTGTCCGGTGGATCTCGATATAGAGACCTTGAAGATGGTCGATGGAAAACCCGTTCTCGTTGTGGAGGATCACAACGTTTTCACAGGACTTGGAAGCTTCCTTGGAACCACCCTTCTTGAAAACGGCATCATCCCGAAGAAATACGTGAGAGTAGGTGTTCCAGAATTCGCCGTGTCCGGCAGTTACACGATGCTCTACAAACTCTACGGCCTGGATAAAGATGGAATAATTTCCAGACTCAGAGAGATGCTCTAA

**> pQR1745**

MERFPYEKLPESELKELKELGRLCRGDILKMTYIANSGHPGGSMSSIDLYLTVFKYAKLRPVDDPARDRIVISHGHTSPGVYAAMARLGFVDLDEVLAGFRHPASVFEGHVTRGVGIIDWTTGNLGQGLSAGLGFALASRFTGKDYHVFVLMSDAEQAKGQVAEARRVAKKYGVTNLTVIIDYNDAQISGRARDVMPVNIKENYLADGWRVIEIDGHDYEQIYLALKEAVEDELNPVAIIAKTVMGKGVSFMENEVKYHGKPLNREELEKALAELGIENDVDVYIEKRKQLPVEKHKKVYKTYPIKIDTGEPITYTSPTDNRSAFGKAILDLVKKNVNNPETTPIVAVDCDLKGSVKLDLDKEFPERLLEVGVQEHNAAAMAGALSAEGVITFFADFGVFGISETYNQHRLNAINGTNLKVVVTHCGLNVGEDGKTHHGLDYVSGPMNWYGFKVIVPGDPNQTDRVVRYAAKEYGNFVIAMGRSKLPIILDENGKPFFGEGYTFEYGKIDVVRKGDDAVIITYGSTLCEAVNAADELKKEGVNVAVLNVSCPVDLDIETLKMVDGKPVLVVEDHNVFTGLGSFLGTTLLENGIIPKKYVRVGVPEFAVSGSYTMLYKLYGLDKDGIISRLREML

### 2.5 TAm DNA/ amino acid sequences

DNA sequences were retrieved from the NCBI database [5] and amino acid sequences were obtained through UniProtKB [6].

**>TAm**_**Gste**_

ATGAAATTGGCAAAACGGGTGGCGTCGCTGACGCCATCGGCGACTTTGGCCATTACGGAGAAAGCAAAAGAACTAAAAGCGGCCGGGCATGACGTGATTGGTCTCGGAGCTGGCGAACCGGATTTCAACACGCCACAGCACATTCTTGATGCCGCCATCAAGGCAATGAACGAAGGACATACGAAATATACACCATCGGGCGGTTTGCCGGCGTTAAAGGAGGAAATTATAAAAAAATTCGCCCGCGACCAAGGCTTGGATTATGAGCCGGCTGAAGTGATTGTATGCGTCGGAGCGAAGCACGCCCTTTACACGCTGTTCCAAGTATTGCTCGATGAAGGCGACGAAGTGATCATTCCGACGCCATACTGGGTGAGCTATCCGGAACAAGTGAAACTGGCGGGCGGTGTTCCGGTTTACGTCGAAGGGCTTGAACAAAATCATTTTAAAATTACGCCGGAGCAGCTGAAACAGGCAATCACGCCGCGGACGAAAGCGGTTATCATCAACTCGCCGAGCAACCCGACTGGCATGATTTATACAGCCGAAGAGTTGAAGGCGCTTGGTGAGGTGTGCCTAGCGCATGGTGTATTGATCGTGTCAGATGAAATTTACGAAAAATTGACTTACGGCGGGGCGAAGCATGTGTCCATCGCTGAGTTGTCGCCGGAGCTGAAGGCGCAGACAGTCATCATTAACGGCGTGTCAAAGTCGCATTCGATGACGGGCTGGCGCATTGGTTATGCGGCGGGGCCGAAAGATATTATTAAGGCAATGACAGATTTGGCGAGCCACAGCACGTCCAACCCGACGTCAATCGCCCAATACGCGGCCATCGCTGCTTACAGCGGGCCGCAGGAGCCGGTCGAACAAATGCGCCAAGCGTTTGAACAACGGCTCAATATCATTTACGACAAGCTCGTGCAAATTCCAGGATTCACGTGCGTTAAGCCACAAGGGGCGTTTTATTTGTTCCCGAACGCCCGCGAAGCGGCTGCAATGGCCGGCTGCCGCACGGTCGACGAGTTCGTCGCTGCCTTGTTGGAGGAAGCGAAAGTCGCGCTTGTGCCCGGCTCTGGGTTTGGAGCGCCGGATAACGTTCGCTTGTCATACGCGACATCGCTCGATGCACTGGAAACCGCCGTGGAACGCATCCACCGGTTTATGGAAGCGCGCGCTTAA

**>TAm**_**Gste**_

MKLAKRVASLTPSATLAITEKAKELKAAGHDVIGLGAGEPDFNTPQHILDAAIKAMNEGHTKYTPSGGLPALKEEIIKKFARDQGLDYEPAEVIVCVGAKHALYTLFQVLLDEGDEVIIPTPYWVSYPEQVKLAGGVPVYVEGLEQNHFKITPEQLKQAITPRTKAVIINSPSNPTGMITAEELKALGEVCLAHGVLIVSDEIYEKLTYGGAKHVSIAELSPELKAQTVIINGVSKSHSMTGWRIGYAAGPKDIIKAMTDLASHSTSNPTSIAQYAAIAAYSGPQEPVEQMRQAFEQRLNIIYDKLVQIPGFTCVKPQGAFYLFPNAREAAAMAGCRTVDEFVAALLEEAKVALVPGSGFGAPDNVRLSYATSLDALETAVERIHRFMEARA

**>TAm**_**DGeo**_

ATGTTCGAGGACACGCCCGCACCCTTTCCACCGCACATTCTGCTGACGCCCGGTCCGACACCGATTCACCCCCGGGCCCAGCGGGCGCTGCTGCGCGAGATGCTGGGGCACATGGACCCTGAGGTGTTCGCCCTGAACCGCGAGATCCAGGCGGACTTGCGGGTGATGTACGGGACGGGGCCCCAGACCTTTACGGCGCTGCTGGCGGGCACCGGGAGCCTGGGCATGGAGGCGGGCTTCGCCAACTTGGTGGAGAGGGGAGACGACGTGCTGATCTGCGTCAATGGTGCCTTTGGTCAGCGCATGGCCGAGATGGCGGCGCGCTACGGTGCGAATGTACGGCGGGTGACCGCGCCGCTGGGCGAGCCGATCGACCCGGCCGACGTGGCTGCGCGGTTGAGCGGCGCGCGGCTGGTGGCGGTGGTGCATGGGGAGACGAGCACGGGTGTGCTCAATCCGCTTCCGGAGATTGCCGAGGCCGTGCGCGGGAGCGGGGCATTGCTGGCCGTGGACGCCGTGACGACCGCCGGGATGGAACCCTTCCATATGGCGGACTGGGGCGTGGACTACGCCTATACCGGCGCGCAGAAGTGCCTCTCGGCACCGCCCGGCCTGGCCCCGGTGGCGATCAGCGACCGTGCTCTCGCTCGCCACGCGGCCCGCCGCACGCCCACGCCGCTGTGGTACTGCGATTTTGAGGGCCTGCGCGACTACTGGGACCGGCACAGCTACCACCACACGGTCCCGGTGAATCTGCACTACGCCTTCCACGCCGCCCTGCGCGCCGCACTCGAAGAAGGCCTCCAAGCCCGGCAGGCCCGCGTGCGCGACCTTGGCCAGGCGGTGCTGGCGGCCCTGACGCCGCTGGGCTTCACGCCGTATGTGGCCGATCCCGCCGCGCGGCTGCCCACCGTCTTGGCCCTGCGTCTTCCTCCCGGCTTCGACGACGCGGGCGTTCGCCAGGCCCTACGGGAACGCGGGATCAGCGTGACCGGCGGCCTGGGACCGACGGCAGGGCTGATCTGGCGTCTGGGCCTGATGGGGGAAGCGGCTCGCCCCGCGCCCTACCGCGCGCTGATGCTCGCCCTGGAAGACCTGCTGGGCGAGCGGGGCTTGGTGGCGCGCTTCGAGGAGGCGCTGGGCGTCGCGGCCTGA

**>TAm**_**DGeo**_

MFEDTPAPFPPHILLTPGPTPIHPRAQRALLREMLGHMDPEVFALNREIQADLRVMYGTGPQTFTALLAGTGSLGMEAGFANLVERGDDVLICVNGAFGQRMAEMAARYGANVRRVTAPLGEPIDPADVAARLSGARLVAVVHGETSTGVLNPLPEIAEAVRGSGALLAVDAVTTAGMEPFHMADWGVDYAYTGAQKCLSAPPGLAPVAISDRALARHAARRTPTPLWYCDFEGLRDYWDRHSYHHTVPVNLHYAFHAALRAALEEGLQARQARVRDLGQAVLAALTPLGFTPYVADPAARLPTVLALRLPPGFDDAGVRQALRERGISVTGGLGPTAGLIWRLGLMGEAARPAPYRALMLALEDLLGERGLVARFEEALGVAA

**>pQR1746**

ATGACCTCTCCTTTCCGCCTCTCCGCCCGCGCCCAGAGCCTCAAGCCGTCTGCGACAGTGGCGGTCACGTCCCGCGCCCTGGAACTCCAGCGTCAGGGCCTGGACGTGATTTCCATGAGCGTGGGCGAGCCGGATTTCGACACGCCGCCACATGTCAAGGCCGCCGGCATCGCCGCCATCGAGGAAGGCAAGACCAAATACACCCCGGTCAGCGGCATTCCCGAACTGCGCGAGGCCATCAGCGCCAAGTTTCGGCGCGAAAACGGCCTGGACTACGCGCCGAACGCCGTGACGGTAACGAGCGGCGGTAAACAGGCGCTGTTCAACGCCTTTTTCGCGTTGCTGAACCCCGGCGACGAGGTGCTGATTCCCGCGCCCCACTGGGTCAGCTACCCCGAAATGGTCGCGCTGACCGGCGCGGTGCCGGTAACCGTACCCACTACGCCGCAGCAGGGCTTTCAACTCGACCCGGACGCCCTCGCCGCCGCCATCACGCCGCGCACCCGCATGGTGATTCTCAACAGCCCCGGCAACCCGACGGGCGCGGTGTTTCCGCCGGAAACCTTGCGGGCGGTGGCCGACCTCGCCACGCAGCACGGCTTGATGATCGTCACCGACGAAATCTACGAGCACCTCGTCTACGACGCCGAGCAGGTCAGCATCGGCACCTACGCGCCGGAGCACACCCTGACCATCAATGGCGCGAGCAAAGCGTATGCCATGACCGGCTGGCGCATCGGCTACGCGGGCGGGCCGCGCGAGGTGATTGCCGCCATGAACGCGCTGCAATCGCAAAGCACCAGCAACGCCAGCAGCGTCAGCCAGTACGCCGCCCTCGCCGCGCTGGAACAGCACGAGGAAACCATGCGCTTCATCGACAGGGCCCGCACCGCCTACCGCGAACGGCGCGACCGCATCGTGGCGGGCCTCAACGCGCTGGGGTTGCCCACGCCCACGCCGCAAGGGGCCTTTTACGTGATGGCCGACACCCGCGCCATTCACACCGACGAACTCGAAGCCGCCCGCATCATTCTGGATGAGGCGCAGGTCGCCGTCGTGCCCGGCACCGATTTCGCCGCGCCGGGACAGGTGCGCCTGAGCTACGCGACCAGCATGGACAACATCGAGGAAGTGCTGCGGCGGCTGGAAGGGGTCGTGCGGCGCTAA

**>pQR1746**

MTSPFRLSARAQSLKPSATVAVTSRALELQRQGLDVISMSVGEPDFDTPPHVKAAGIAAIEEGKTKYTPVSGIPELREAISAKFRRENGLDYAPNAVTVTSGGKQALFNAFFALLNPGDEVLIPAPHWVSYPEMVALTGAVPVTVPTTPQQGFQLDPDALAAAITPRTRMVILNSPGNPTGAVFPPETLRAVADLATQHGLMIVTDEIYEHLVYDAEQVSIGTYAPEHTLTINGASKAYAMTGWRIGYAGGPREVIAAMNALQSQSTSNASSVSQYAALAALEQHEETMRFIDRARTAYRERRDRIVAGLNALGLPTPTPQGAFYVMADTRAIHTDELEAARIILDEAQVAVVPGTDFAAPGQVRLSYATSMDNIEEVLRRLEGVVRR

**>pQR1748**

ATGACTGACCGACCTCGTATCTCCGCACGCATCGGCGGTATCTCCGAGTCAGCGACCCTGGCGGTGGACGCCAAGGCCAAGGCCCTGAAGGCCGCTGGGCATCCCGTGATCGGCTTCGGCGCCGGGGAGCCTGACTTCCCCACGCCCGACTACATCGTGGAGGCAGCGGTCGCCGCCTGCCGCGACTCGCGCTTCCACCGCTACACCCCGGCGGGAGGCCTCCCCGAACTCAAGGAAGCCATCGCGGCTAAGACGCTGCGCGACTCCGGCTACCGGGTGGAGCCGAACCAAGTCCTGGTCACCAACGGCGGCAAGCAAGCGATCTACGAGGCGTTCGCCACGCTGCTCGATCCGGGCGACGAGGTCATCGTGATCGCGCCCTACTGGACCACCTACCCTGAATCGATCCGGCTGGCCGGAGGAACCCCGGTCTACGTGGTCACCGACGAGTCCACTGGCTACCTGGCCACGGTCGAGCAGCTGGAGGCGGCCCGCACCGACCGCACCAAGGTGCTGCTGTTCGTCTCCCCCTCGAACCCGACCGGCGCGGTGTACTCGCCCGAGCAGGTCCGCGAGATCGGCCGGTGGGCCCTCGAACACAACCTGTGGGTGCTCACCGACGAGATCTACGAGCACCTCGTCTACGGGGACGCCCGGTTCTCCTCGATGCCGGTGGAAGTTCCGGAACTGGCCGACCGCACCGTGGTGGTCAACGGGGTGGCCAAGACCTACGCCATGACCGGGTGGCGGGTCGGCTGGCTCATCGGCCCCGTGGACGTGGTCAAGGCTGCGACCAACCTGCAGTCGCACGCCACCTCCAATGTGGCCAACGTCTCGCAGGCCGCGGCTCTGGCAGCGGTCTCCGGCGACCTGTCGGCCGTGGAGGAGATGAAGCAGGCCTTCGACCGGCGGCGGCAGACCATTGTGCGGATGCTCAACGAGATCCCCGGTGTGGTGTGCCCCGAGCCCCAGGGCGCGTTCTACGCCTACCCGTCGGTCAAGGAGATCCTCGGCAAGGAGATCCGCGGTCAGCGTCCGCAGACCTCCAGCGAGCTGGCGTCGCTGATCCTGGAGCACGCCAAGGTCGCGGTGGTCCCGGGCGAGGCGTTCGGCACTCCGGGCTACCTGCGGTTGTCCTACGCGTTGAGCGACGCCGATCTGGTCGAAGGGGTCAGCCGGATCGCCAAGCTGCTGAGCGAAGCCCACTGA

**>pQR1748**

MTDRPRISARIGGISESATLAVDAKAKALKAAGHPVIGFGAGEPDFPTPDYIVEAAVAACRDSRFHRYTPAGGLPELKEAIAAKTLRDSGYRVEPNQVLVTNGGKQAIYEAFATLLDPGDEVIVIAPYWTTYPESIRLAGGTPVYVVTDESTGYLATVEQLEAARTDRTKVLLFVSPSNPTGAVYSPEQVREIGRWALEHNLWVLTDEIYEHLVYGDARFSSMPVEVPELADRTVVVNGVAKTYAMTGWRVGWLIGPVDVVKAATNLQSHATSNVANVSQAAALAAVSGDLSAVEEMKQAFDRRRQTIVRMLNEIPGVVCPEPQGAFYAYPSVKEILGKEIRGQRPQTSSELASLILEHAKVAVVPGEAFGTPGYLRLSYALSDADLVEGVSRIAKLLSEAH

**>pQR1749**

ATGGTATCCAGGAGAATATCAGAGATTCCCATATCGAAAACCATGGAACTCGACGCGAAGGCCAAAGCCCTCATAAAAAAGGGAGAAGACGTGATCAATCTAACGGCTGGTGAGCCGGATTTTCCCACACCGGAACCCGTCGTGGAAGAAGCGGTGAGATTTCTCCAGAAAGGAGAAGTGAAATACACAGATCCTCGTGGTATCTACGAACTCAGAGAGGGTATAGCGAAAAGGATAGGCGAGAGATACAAAAAAGATATCTCACCGGATCAGGTCGTGGTGACGAATGGAGCGAAACAGGCTCTGTTCAATGCTTTCATGGCCCTTCTCGATCCCGGTGACGAAGTGATCGTGTTTTCTCCCGTCTGGGTCAGCTACATTCCTCAGATCATCCTTGCTGGTGGCACGGTGAACGTGGTTGAGACGTTCATGAGTAAAAATTTCCAGCCCAGTCTGGAAGAGGTGGAAGGGCTTCTTGTTGGGAAAACGAAAGCCGTTCTTATCAACTCGCCGAACAATCCCACTGGTGTGGTGTACAGAAGAGAGTTCCTTGAAGGACTTGTGAGACTTGCCAAGAAGAGGAATTTCTACATAATCAGCGACGAAGTCTACGATTCCCTTGTTTACACGGATGAATTCACATCGATACTCGATGTTTCTGAAGGATTCGACCGGATAGTTTACATAAACGGCTTCTCGAAGTCTCACTCCATGACCGGCTGGAGGGTGGGTTACCTGATATCGAGCGAAAAAGTAGCGACCGCTGTGTCGAAGATCCAGTCTCACACCACCTCCTGTATCAACACGGTAGCACAGTACGCCGCCTTGAAGGCTCTGGAAGTGGACAACTCTTACATGGTTCAGACCTTTAAAGAAAGAAAAAATTTCGTGGTGGAAAGATTGAAAAAGATGGGTGTTAAGTTCGTGGAACCAGAAGGTGCGTTCTACCTCTTTTTCAAAGTCCGGGGTGACGATGTGAAATTCTGTGAAAGGCTCCTCGAAGAAAAGAAGGTTGCACTCGTTCCAGGATCCGCTTTTCTGAAGCCTGGATTTGTGAGGCTTTCTTTTGCCACATCTATAGAAAGACTTACGGAGGCGCTGGATAGAATTGAAGACTTCCTCAATTCTCGTTGA

**>pQR1749**

MVSRRISEIPISKTMELDAKAKALIKKGEDVINLTAGEPDFPTPEPVVEEAVRFLQKGEVKYTDPRGIYELREGIAKRIGERYKKDISPDQVVVTNGAKQALFNAFMALLDPGDEVIVFSPVWVSYIPQIILAGGTVNVVETFMSKNFQPSLEEVEGLLVGKTKAVLINSPNNPTGVVYRREFLEGLVRLAKKRNFYIISDEVYDSLVYTDEFTSILDVSEGFDRIVYINGFSKSHSMTGWRVGYLISSEKVATAVSKIQSHTTSCINTVAQYAALKALEVDNSYMVQTFKERKNFVVERLKKMGVKFVEPEGAFYLFFKVRGDDVKFCERLLEEKKVALVPGSAFLKPGFVRLSFATSIERLTEALDRIEDFLNSR

**>pQR1751**

ATGGCACCTGACCTGCGCCACCTGCACACCTTCGGCGAACTGGATCCGCCGCAACGCCTGTTGATGGGCCCCGGCCCGGTCAATGCGCATCCACGCGTGCTGCGTGCGATGGCGGCCGACCTGCTTGGCCAGTTCGACCCGGAAATGACCACCTACATGAACGAGGTGATGGCGCTGTACCGCCCCTTGTTCGGCACCCAGAACCGCTGGACCTTTCTGGTCGATGGCACGGCGCGCGCCGGCATCGAAGCCGCGCTGGTGTCGCTGGTGCAGCCGGGCGACCGTGTGCTGGTGATCAACTTCGGCCGCTTCGGTTTGTTGCTGACCGAAATCCTTGGCCGGCTCGGCGCCGACGTCCACACCGTGGATGCGCCGTGGGGCGAGGTGGTGCCGCTGGCGGCGATTGCCGAGGCGATCGCAAGCGTGGCACCCAAGCTGGTGGCCACCGTGCACGGCGACACCTCCACCACCATGGCGCAGCCGCTCGATGGCCTAGGCGCGCTATGCCGGGCGGCCGGCGCGCTGAGTTACGTAGACGCCACAGCCACCATCGGCGGCATGGACATCGCCAGCGACCGCTGGGAGGTGGACGTGGTCACCGCGGGGCTGCAGAAATGCCTGGGCGGGCCGTCCGGCTCGGCGCCGATCACTGTCTCTGCCGCGGCAGCGGAGGCGATCTTTGCGCGGCGGCATGTCGAACGCGGCATCGTGCGCGAGGACATCGCCAACGGCAGCGGCCCACGCATCGCCTCGAATTATTTCGACCTGGCGATGATCATGGATTACTGGTCCGACAAGCGCCTCAATCACCACACCGAAGCCACCACCATGCTGTACGGCGCGCGCGAATGCGCACGCGTGGCCTTGCAGGAAGGCCTGGAGGCGCGCTACGCCCGGCATGCGGCTGCCGGCCGCGCGGTCAGCGCCGGCGTGCGCGCACTGGGGCTGGAGGTGTTCGGCGACGATGCGCACCGCATGAGCAATGTCACCGGCGTGGTGATCCCGCACGGCGTCGACAGTGAAGCAGTGCGGCGGCGCATGCGCGAGGATTTCGAAATCGAGATCGGCACCGCGTTCGGCCCGCTGCAAGGCAGGATCTGGCGCATCGGTGCGATGGGCTACAACGCGATGAAGCACAAGGTGCTGCTCACCCTGGCCGCACTGGAAGCGGTGCTGCGCGCCGAGGGCTACGCGTGCACCCAAGGCCTGGCGGTCGAAGCCGCACGCGCCGCCTGGCATGCGGAGCCGGCTGCATGA

**>pQR1751**

MAPDLRHLHTFGELDPPQRLLMGPGPVNAHPRVLRAMAADLLGQFDPEMTTYMNEVMALYRPLFGTQNRWTFLVDGTARAGIEAALVSLVQPGDRVLVINFGRFGLLLTEILGRLGADVHTVDAPWGEVVPLAAIAEAIASVAPKLVATVHGDTSTTMAQPLDGLGALCRAAGALSYVDATATIGGMDIASDRWEVDVVTAGLQKCLGGPSGSAPITVSAAAAEAIFARRHVERGIVREDIANGSGPRIASNYFDLAMIMDYWSDKRLNHHTEATTMLYGARECARVALQEGLEARYARHAAAGRAVSAGVRALGLEVFGDDAHRMSNVTGVVIPHGVDSEAVRRRMREDFEIEIGTAFGPLQGRIWRIGAMGYNAMKHKVLLTLAALEAVLRAEGYACTQGLAVEAARAAWHAEPAA

**>pQR1752**

ATGGGAAAGTTTCTTAAGAAACACTACATAATGGCACCTGGACCAACACCAGTCCCAAACGATATTTTAACAGAAGGAGCGAAGGAAACAATACACCACAGAACACCTCAGTTTGTTTCCATAATGGAAGAGACCCTCGAAAGTGCAAAGTACATCTTTCAGACAAAACACAACGTGTACGCCTTTGCTTCCACAGGAACTGGCGCTATGGAAGCGGCGGTGGCGAATCTTGTGAGCCCTGGAGACAAAGTGATCGTGGTTGTGGCTGGAAAGTTCGGTGAAAGATGGAGAGAGCTCTGTCAGGCTTACGGTGCTGATATCGTAGAAATCGCCCTCGAATGGGGAGACGCGGTCACACCTGAACAGATCGAAGAGGCTCTCAACAAAAACCCCGATGCGAAGGTCGTCTTCACCACCTACAGTGAAACATCGACGGGTACAGTCATAGACCTCGAAGGAATTGCCAGAGTCACGAAGGAAAAAGACGTTGTTCTTGTGACAGACGCTGTCAGCGCTCTTGGAGCAGAACCACTGAAGATGGATGAATGGGGTGTGGATCTCGTTGTCACAGGTTCACAGAAGGGTTTGATGTTACCTCCAGGACTGGCGCTCATCTCTCTCAACGACAAAGCGTGGGGGCTCGTGGAAAAATCCAGATCTCCAAGGTACTACTTCGATCTGAGGGCCTACAGGAAATCTTACCCCGACAATCCTTACACCCCCGCAGTAAACATGATATACATGTTGAGAAAGGCTCTTCAGATGATAAAAGAGGAAGGCATAGAAAACGTATGGGAAAGGCACAGAATACTGGGAGACGCAACAAGAGCAGCGGTGAAAGCACTTGGACTGGAACTCCTCTCGAAAAGACCGGGAAACGTTGTAACAGCCGTGAAAGTGCCTGAGGGCATCGATGGAAAACAGATTCCCAAGATCATGAGAGACAAGTACGGTGTGACCATCGCCGGTGGACAGGCTAAACTCAAGGGAAAAATATTCAGGATAGCACACCTCGGATACATGTCACCTTTCGACACCATAACTGCCATTTCCGCTCTTGAATTAACCTTGAAGGAACTCGGTTATGAGTTCGAACTCGGAGTCGGTGTTAAGGCAGCCGAAGCTGTCTTCGCTAAAGAATTCATTGGGGAGTGA

**>pQR1752**

MGKFLKKHYIMAPGPTPVPNDILTEGAKETIHHRTPQFVSIMEETLESAKYIFQTKHNVYAFASTGTGAMEAAVANLVSPGDKVIVVVAGKFGERWRELCQAYGADIVEIALEWGDAVTPEQIEEALNKNPDAKVVFTTYSETSTGTVIDLEGIARVTKEKDVVLVTDAVSALGAEPLKMDEWGVDLVVTGSQKGLMLPPGLALISLNDKAWGLVEKSRSPRYYFDLRAYRKSYPDNPYTPAVNMIYMLRKALQMIKEEGIENVWERHRILGDATRAAVKALGLELLSKRPGNVVTAVKVPEGIDGKQIPKIMRDKYGVTIAGGQAKLKGKIFRIAHLGYMSPFDTITAISALELTLKELGYEFELGVGVKAAEAVFAKEFIGE

**>pQR1755**

ATGACTCAGATTTTTAATTTTAGCGCCGGTCCAGCAATGCTGCCGGTTGAAGTACTGCGTCGTGCTGAACAGGAATTGTGTAATTGGAATGGCCTGGGCACATCGGTTATGGAAATCAGCCACCGCAGTAAAGAGTTTATGCAGGTTGCCGCTGAATCCGAACAGGATCTGCGTGATTTGCTGAAAATCCCCTCCAACTACAAAGTGCTCTTTTGCCACGGCGGTGCTCGTGCGCAATTCGCCGCAGTGCCGTTAAATCTTCTGGGCGAACGCTCAACGGCCGACTACATCGACGGCGGGTATTGGGCGCACAGCGCAATCAATGAAGCAGAAAAATACTGCACGCCTAACGTGATTGACGTGAAAATGCGCGTGGGCGAACTGCGTGGCATTAAGCCGATGCGTGAATGGAAATTGTCTGATGACGCGGCGTTTGTGCATTACTGCCCGAATGAAACCATCGACGGTATTGCGATCGAAGAAGAGCCGGACTTTGGCGATAAAATTGTGGTCGCCGACTATTCTTCCAGCATCCTGTCTCGTCGTATTGATGTCAGCCGTTACGGCGTGATCTATGCCGGTGCGCAGAAAAATATCGGCCCTGCCGGCCTGACGCTGGTTATCGTACGTGAAGATTTGCTGGGCAAGGCGCGCCGTGAGCTGCCATCGATTCTGGATTACCAGGTTCTGGCGGACAATGACTCCATGTTTAACACGCCACCGACCTTTGCCTGGTACCTGTCCGGTATGGTCTTCAAATGGCTGAAAGAGTACGGCGGTCTGGCTGAAATGGAAAAACGTAACCAGGAGAAGGCTGACCTGCTGTATAGCGCGATTGACGGTAACGATTTCTATCGTAATGACGTTGCGGTAGCGAACCGTTCTCGCATGAATGTGCCATTCCTGTTGGCGGATTCTGCGCTGGATAAAGTCTTCCTGGAAGAATCAGTCGCTGCAGGTCTGCACGCGCTGAAAGGCCATCGCGTAGTAGGCGGCATGCGTGCCTCTATCTACAATGCGATGCCGTTGGAAGGCGTGAAAGTGCTGACGGAATTTATGGCTGACTTCGCTCGTCGCCACGGTTGA

**>pQR1755**

MTQIFNFSAGPAMLPVEVLRRAEQELCNWNGLGTSVMEISHRSKEFMQVAAESEQDLRDLLKIPSNYKVLFCHGGARAQFAAVPLNLLGERSTADYIDGGYWAHSAINEAEKYCTPNVIDVKMRVGELRGIKPMREWKLSDDAAFVHYCPNETIDGIAIEEEPDFGDKIVVADYSSSILSRRIDVSRYGVIYAGAQKNIGPAGLTLVIVREDLLGKARRELPSILDYQVLADNDSMFNTPPTFAWYLSGMVFKWLKEYGGLAEMEKRNQEKADLLYSAIDGNDFYRNDVAVANRSRMNVPFLLADSALDKVFLEESVAAGLHALKGHRVVGGMRASIYNAMPLEGVKVLTEFMADFARRHG
