# Supplementary material for: Data on a thermostable enzymatic one-pot reaction for the production of a high-value compound from l-arabinose
Source: Data Brief. 2018 May 31;19:1341–54. doi: 10.1016/j.dib.2018.05.140 (PMC6140825; doi:10.1016/j.dib.2018.05.140)
Supplement: Supplementary file 1 — Supplementary material [file mmc1.docx]

Conflict of Interest

All the authors confirms no conflict of Interest
